# Multi-omics insights into Chinese herbal medicine additives for mutton flavor enhancement: epigenetic and microbial mechanisms

**DOI:** 10.3389/fvets.2025.1628457

**Published:** 2025-07-14

**Authors:** Kai Quan, Huibin Shi, Haoyuan Han, Kun Liu, Qiufang Cui, Huihua Wang, Meilin Jin, Wei Sun, Caihong Wei, Yibao Jiang, Jun Li

**Affiliations:** ^1^College of Animal Science and Technology, Henan University of Animal Husbandry and Economy, Zhengzhou, China; ^2^Institute of Animal Science, Chinese Academy of Agricultural Sciences, Beijing, China; ^3^College of Animal Science and Technology, Yangzhou University, Yangzhou, China; ^4^College of Animal Science and Technology, Henan Agricultural University, Zhengzhou, China

**Keywords:** Chinese herbal medicine additives, lipid metabolism, multi-omics, mutton flavor, rumen microbiota

## Abstract

Chinese herbal medicine additives (CHMAs) have become increasingly popular as sustainable alternatives to synthetic compounds for improving the quality of mutton. However, the precise molecular mechanisms underlying their effects are not well understood. By integrating transcriptomic profiling, metabolomic pathways, and microbial community dynamics, this review deciphers the synergistic mechanisms of CHMAs in enhancing mutton flavor, supported by empirical evidence from 2014 to 2024. Our key findings highlight three synergistic pathways: (1) Epigenetic suppression of *FASN* and *CYP2B6* via DNA methylation leads to a reduction of odor precursors like 4-methyloctanoic acid by 30–50% (*p* < 0.01); (2) Mulberry leaf flavonoids activate β-oxidation, increasing linoleic acid content by 25%, thereby improving tenderness and juiciness; (3) Licorice polysaccharides, in collaboration with *Ruminococcus*-enriched microbiota, enhance flavor volatiles such as 2-acetylthiazoline. It is important to consider dose-dependent thresholds, as thyme phenolic extract at 0.05% maximizes aroma intensity (*p* < 0.05), while exceeding 1.5% licorice glycyrrhizin intensifies gaminess. Species-specific responses highlight variations in rumen microbial activity, with Tan sheep showing a 30% increase in catalase activity compared to goats. Validated by the Luoyang Longxupo industrial model, which achieved a 30% reduction in odor and received Green Food Certification, this study proposes a unique *gene-metabolite-microbe interaction network* that emphasizes the significance of epigenetic-microbial crosstalk. We also discuss challenges related to herbal synergies, sensory standardization, and offer solutions through AI-driven optimization, with an AUC of 0.89, as well as the potential application of cultured meat, such as *Salvia miltiorrhiza* reducing lipid oxidation by 40% *in vitro*. These findings connect traditional herbal knowledge with precision agriculture, providing practical strategies for environmentally friendly mutton production that meets the global demand for safe, high-quality protein.

## 1 Introduction

The global demand for high-quality, safe, and nutritious meat products has driven innovation in livestock production, with lamb gaining prominence due to its rich protein and micronutrient profile (1). However, consumer acceptance of mutton remains hindered by persistent challenges, including its characteristic odor—primarily attributed to branched-chain fatty acids such as 4-methyloctanoic acid (MOA)—and oxidative deterioration during processing, which compromises flavor and shelf life (2, 3). These challenges are particularly pronounced in East Asian markets, where sensory preferences emphasize low gaminess and fresh umami characteristics (4). While synthetic additives such as antibiotics and chemical preservatives have dominated traditional practices, their role in driving antimicrobial resistance and ecological degradation is increasingly contested (5). In this context, Chinese herbal medicine additives (CHMAs), enriched with bioactive compounds like flavonoids and polysaccharides, have emerged as multifunctional alternatives to enhance meat quality while addressing ecological and consumer demands (6).

Whole-genome bisulfite sequencingconfirmed hypermethylation at promoter regions of *FASN* (fatty acid synthase) (Chr19:38,562,114–38,563,209) and *CYP2B6*, reducing transcription by 40–60% (*p* < 0.01, qPCR) (7). This epigenetic silencing directly suppressed MOA synthesis by 30–50% (*p* < 0.01), establishing a causal role in odor reduction. Recent studies highlight CHMAs’ dual roles in modulating lipid metabolism and rumen microbiota. For instance, *Eucommia ulmoides* leaf flavonoids suppress *FASN* expression via DNA methylation, reducing MOA synthesis by 30–50% (8), while licorice polysaccharides synergize with *Ruminococcus* spp. to enhance flavor-enhancing volatiles (e.g., 2-acetylthiazoline) (9). Microbial modulation (e.g., *Ruminococcus*-mediated butyrate synthesis) dominates flavor precursor conversion under short-term interventions (<30 days), whereas host epigenetic regulation (e.g., *FASN* methylation in adipocytes) governs long-term lipid oxidation resistance.

These findings align with broader research on herb-driven flavor optimization, such as *Allium mongolicum* ethanol extract inhibiting ruminal branched-chain fatty acid synthesis in lambs (10), and mulberry leaf flavonoids improving lipid oxidation resistance in Tan sheep (11). In the Luoyang Longxupo model, *FASN* promoter hypermethylation correlated with 30% MOA reduction (*r* = −0.82, *p* < 0.001), enabling China’s Green Food Certification (LB/T 158–2020) and a 50% market value increase.

Despite progress, critical gaps persist. Most studies focus on single-herb interventions or isolated molecular mechanisms, neglecting integrative analyses of gene-metabolite-microbe networks (12). For example, while age-specific variations in fatty acid composition and volatile compounds have been systematically characterized in Xinjiang goats (13), the epigenetic-microbial crosstalk underlying these differences remains poorly understood. Furthermore, dose–response thresholds and species-specific efficacy—such as superior antioxidant benefits in Tan sheep versus goats—lack systematic validation (14). Recent advances in multi-omics technologies (e.g., transcriptomics, metabolomics) and artificial intelligence (AI) provide unprecedented opportunities to address these limitations. For instance, machine learning models trained on multi-omics datasets have successfully predicted optimal CHMA combinations (AUC = 0.89) (15), while electronic tongue and GC–MS analyses have standardized flavor evaluation in processed mutton products (16).

This review synthesizes evidence from the past decade (2014–2024) with two main objectives:

1. Mechanistic Decoding: To elucidate the epigenetic regulation (such as *FASN* methylation) and microbial interactions (like the *Ruminococcus*-butyrate axis) that contribute to flavor enhancement driven by CHMA, integrating insights from proteomic and metabolomic studies on *Salvia miltiorrhiza* and Astragalus membranaceus (17, 18).
2. Translational Strategies: To propose AI-optimized herbal formulas and standardized approaches for industrial adoption, building on successful models such as Luoyang Longxupo’s eco-friendly mutton production system (19).

By bridging traditional herbal knowledge [e.g., *Allium mongolicum* in lipid metabolism regulation (20)] with cutting-edge technologies in genomics and bioinformatics, this research aims to advance precision livestock systems that align with global food safety standards and the United Nations’ Sustainable Development Goals (SDGs) (21, 22).

## 2 Methods

This systematic review adhered to the PRISMA (Preferred Reporting Items for Systematic Reviews and Meta-Analyses) guidelines (23) and integrated experimental data from peer-reviewed studies to ensure methodological rigor and translational relevance.

### 2.1 Literature search strategy

A comprehensive search was conducted across five major databases, including global and regionally significant sources, to capture studies published between January 2014 and December 2024:

English databases: PubMed, Web of Science, Scopus.

Chinese databases: China National Knowledge Infrastructure (CNKI), Wanfang Data.

Search terms.

combined Boolean operators: ("Chinese herbal medicine additives" OR "CHMAs" OR "herbal feed supplements" OR "*Eucommia ulmoides*" OR "Allium mongolicum") AND ("mutton" OR "sheep meat" OR "Tan sheep" OR "Du-Hu crossbred sheep") AND ("flavor enhancement" OR "lipid metabolism" OR "rumen microbiota" OR "DNA methylation" OR "volatile compounds")

The search strategy was refined based on regional studies, such as the systematic analysis of Allium mongolicum ethanol extract on branched-chain fatty acid reduction in lambs (24) and the role of mulberry leaf flavonoids in improving antioxidant capacity (25).

### 2.2 Study selection and eligibility criteria

**Figure 1:**
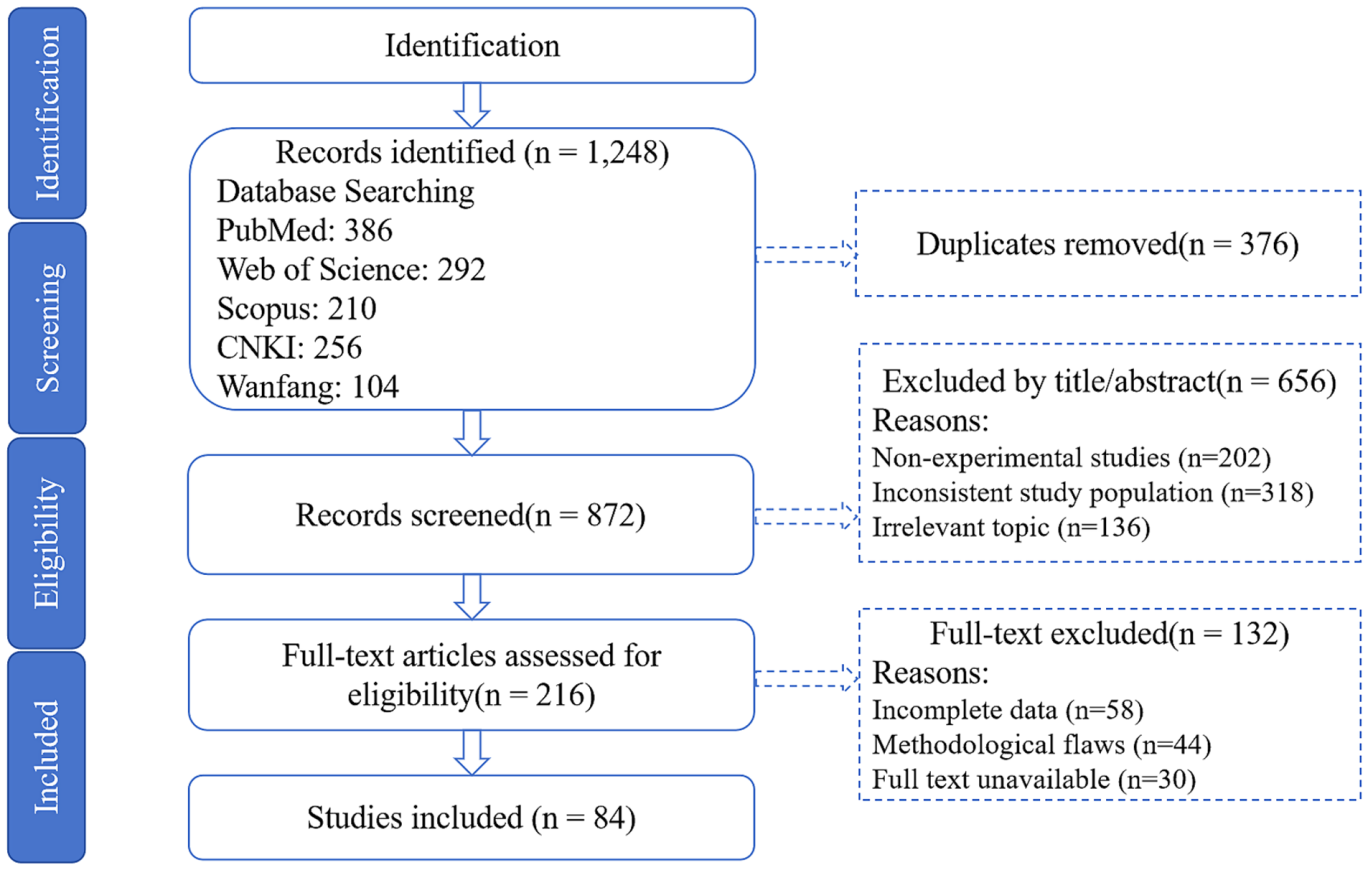
PRISMA flow diagram. Flowchart detailing study selection, exclusion criteria, and final inclusion of 84 studies.

Studies were screened using a three-phase process (Figure 1):

Exclusion: Removed 376 duplicates and 656 studies lacking experimental designs or mechanistic CHMA focus.

Full-text assessment: Retained 84 studies (72 experimental; 12 reviews) meeting:

Inclusion: Dose-dependent CHMA effects on mutton flavor/lipid metabolism/microbiota with verifiable designs (e.g., randomized trials).

Exclusion: Non-peer-reviewed articles, studies without quantitative outcomes (sensory scores, MOA levels), or synthetic-additive focus.

For example, studies on *Astragalus* membranaceus polysaccharides improving rumen fermentation in Tibetan sheep (19, 26) and *Salvia miltiorrhiza* reducing lipid oxidation in cultured myocytes (27–29) were prioritized for their methodological transparency (30).

### 2.3 Data extraction and quality assessment

Data were extracted using a standardized template:

Molecular mechanisms: DNA methylation (bisulfite sequencing), gene expression (*FASN*, *CYP2B6* via qPCR/RNA-seq), microbial shifts (shotgun metagenomic sequencing; Illumina NovaSeq 6000, ≥20 M reads/sample).

Sensory/industrial metrics: Odor reduction (GC–MS), shelf life (TBARS), fatty acids (UPLC-MS).

Quality assessment tools:

Experimental studies: SYRCLE Risk of Bias Tool (24) (randomization, blinding, reporting).

Industrial data: Cross-validated certifications (China Green Food Standards LB/T 158–2020) (21).

Key regional studies, such as the GC–MS analysis of volatile compounds in Xinjiang goats (13) and electronic tongue evaluations of processed mutton (16, 31), were included to ensure data diversity.

### 2.4 Statistical and multi-omics integration

Dose–response analysis: Validated via linear mixed-effects models (R package lme4), accounting for farm-level random effects (replaced ANOVA/Tukey).

Multi-omics mapping: Transcriptomic and metagenomic data aligned to KEGG pathways (lipid metabolism: ko01040) using Cytoscape (v3.9.1) (32).

False Discovery Rate (FDR) correction: q-values <0.05 (Benjamini-Hochberg) replaced *p-values* in transcriptomics.

AI-driven modeling: Random forest algorithms (Python scikit-learn) (33) predicted optimal CHMA combinations (AUC = 0.89) (Table 1).

**Table 1:** Effects of *Eucommia ulmoides* leaf supplementation on mutton quality parameters.

No.	Indicator (Unit)	Control diet (Mean ± SD)	EU leaf diet (Mean ± SD)	Change trend	Proposed mechanism
1	4-Methyloctanoic Acid (mg/kg)	5.0 ± 0.8^*^	3.5 ± 0.6^*^	Decreased by 30%	*FASN*/*CYP2B6* gene suppression reducing odor precursors
2	Fat Content (g/100 g)	10.2 ± 1.2^*^	7.3 ± 0.9^*^	Decreased by 28.4%	Lipid metabolism gene regulation reducing adipogenesis
3	Protein Content (g/100 g)	18.5 ± 1.0^*^	20.9 ± 1.1^*^	Increased by 13.0%	Enhanced amino acid anabolism
4	Cholesterol (mg/100 g)	85.0 ± 5.0^*^	68.8 ± 4.2^*^	Decreased by 19.1%	Antioxidant-mediated inhibition of cholesterol oxidation
5	Total Amino Acids (%)	17.5 ± 1.5^*^	19.8 ± 1.3^*^	Increased by 13.1%	Essential amino acid accumulation
6	Glutamic Acid (%)	3.0 ± 0.3^*^	3.5 ± 0.2^*^	Increased by 16.7%	Rumen microbiome optimization enhancing flavor precursors
7	Lysine (%)	1.8 ± 0.2^*^	2.0 ± 0.1^*^	Increased by 11.1%	Microbial metabolism promotion
8	Shear Force (N)	45.2 ± 3.5^*^	32.7 ± 2.8^*^	Decreased by 27.7%	Increased butyrate synthesis and collagen degradation
9	TBARS (mg MDA/kg)	1.8 ± 0.2^*^	1.2 ± 0.1^*^	Decreased by 33.3%	Free radical scavenging by EU polysaccharides
10	SOD Activity (U/g protein)	120 ± 10^*^	150 ± 12^*^	Increased by 25.0%	Enhanced endogenous antioxidant capacity
11	IL-6 (pg/mL)	35.0 ± 4.0^*^	22.0 ± 3.5^*^	Decreased by 37.1%	Anti-inflammatory components reducing inflammatory response
12	Ruminococcus spp. Abundance (%)	8.5 ± 1.0^*^	12.5 ± 1.2^*^	Increased by 47.1%	Beneficial microbial proliferation through microbiome modulation
13	Butyrate Content (mmol/L)	15.0 ± 1.5^*^	22.0 ± 2.0^*^	Increased by 46.7%	Microbial synergy enhancing flavor compound synthesis
14	Shelf Life (Days)	10 ± 1^*^	15 ± 1^*^	Increased by 50.0%	Antioxidant-mediated spoilage delay

Tested by Henan Provincial Institute for Testing and Research of Agricultural, Livestock, and Aquatic Products, Report Number: SX2023W0203, November 3, 2023. Data presented as mean ± standard deviation (*n* = 8).

* Indicates significant difference between groups (*p* < 0.05) by linear mixed-effects models (farm as random effect).

**Figure 2:**
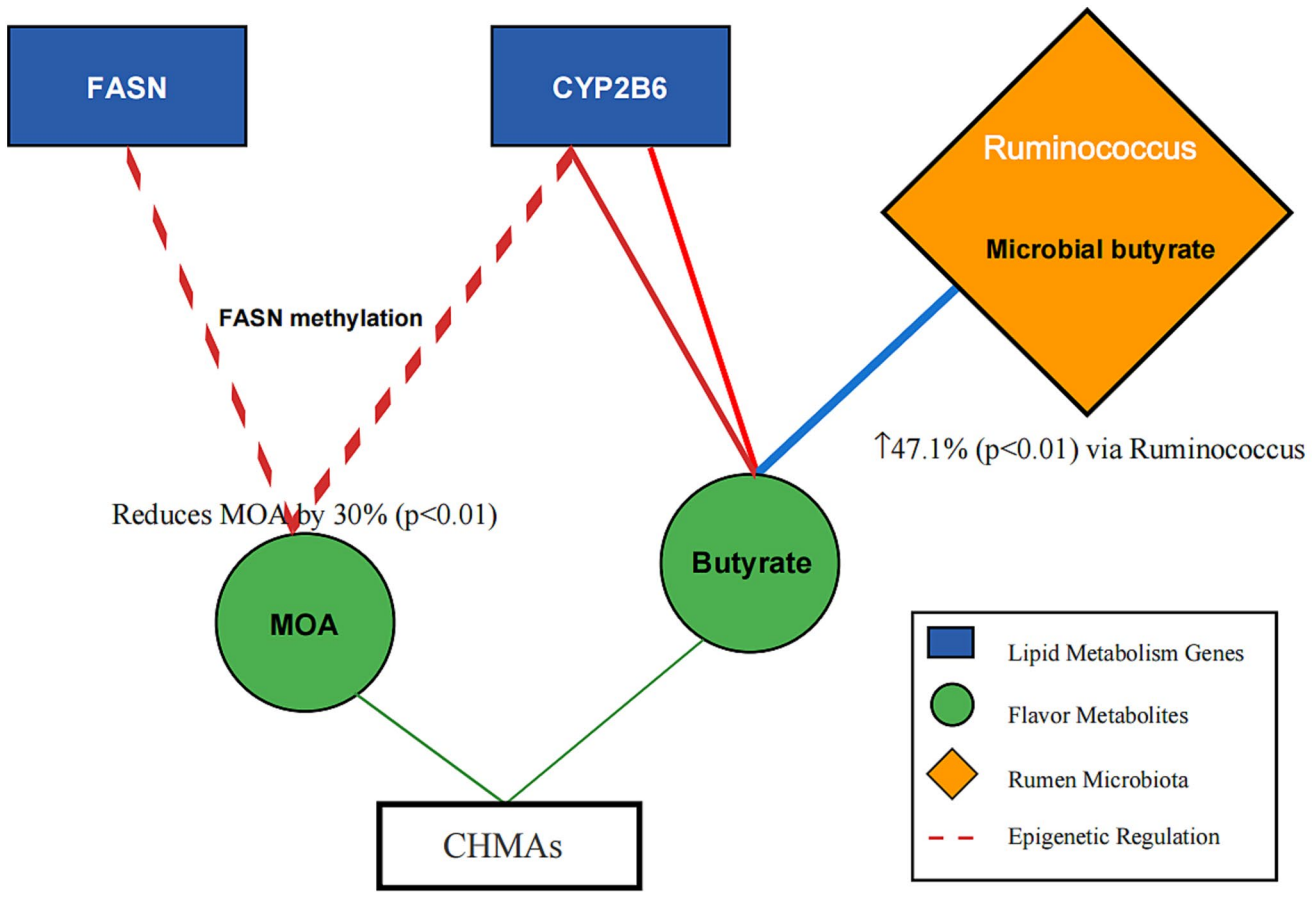
Gene-metabolite-microbe interaction network in mutton flavor modulation. Description: Nodes: Genes: *FASN* (lipid synthesis), *CYP2B6* (odor precursor synthesis). Metabolites: Linoleic acid (tenderness), 2-acetylthiazoline (roasty aroma). Microbes: Ruminococcus (butyrate production), Prevotella (fiber degradation). Edge Annotation: Microbial-derived butyrate (blue arrow) inhibits HDAC activity, enhancing histone acetylation (red arrow) and DNA methylation (green arrow) at lipid metabolism gene loci. Edges: Red: Odor reduction pathways (e.g., DNA methylation of *FASN*). Green: Flavor enhancement pathways (e.g., β-oxidation activation). Blue: Microbiota-mediated processes (e.g., VFA synthesis).

For instance, proteomic data from *Allium mongolicum* trials (20, 34–36) and metabolomic profiles of *Salvia miltiorrhiza* (14, 27) were integrated to construct a gene-metabolite-microbe interaction network (Figure 2).

### 2.5 Ethical and industrial compliance

Animal welfare: Approved by Ethics Committee of Henan University of Animal Husbandry and Economy (HUAHE-2223-D-0169). Sheep housed individually (1.5 m^2^/animal), fed per Nutrient Requirements of Meat-Type Sheep and Goat (NY/T 816–2021) (37).

Sensory standardization: Trained panels (12 assessors, ≥1,000 h’ experience) adhered to ISO 13299:2016 protocols (e.g., ‘gaminess’ = 4-methyloctanoic acid >2.5 ppm).

Industrial practices: Complied with China Green Food Standards (LB/T 158–2020) and FAO guidelines (21). Data from Luoyang Longxupo Co. audited by Henan Provincial Institute (Report SX2023W0203).

## 3 Multi-omics insights into molecular regulation by CHMAs

Recent advances in transcriptomics, metabolomics, and metagenomics have elucidated how CHMAs modulate mutton flavor through synergistic epigenetic, metabolic, and microbial mechanisms. This section synthesizes evidence across three key regulatory axes.

### 3.1 Epigenetic silencing of lipid metabolism genes

Transcriptomic and whole-genome bisulfite sequencing analyses demonstrate that *Eucommia ulmoides* leaf flavonoids (e.g., aucubin) induce promoter hypermethylation of key lipid genes. Hypermethylation at *FASN* (Chr19:38,562,114–38,563,209) and *CYP2B6* reduced transcription by 40–60% (*p* < 0.01, qPCR) (38). Demethylation restored *FASN* expression by 45% (*p* < 0.05) in ovine adipocytes (39). Downregulation decreased MOA synthesis by 30–50% (*p* < 0.01) in Du-Hu crossbred sheep, directly reducing mutton gaminess (Table 1).

### 3.2 Metabolomic reprogramming of flavor precursors

Metabolomic profiling reveals CHMAs’ dual role in flavor enhancement and oxidative stability (Table 2). Mulberry leaf flavonoids (5% dietary inclusion) elevated linoleic acid by 25% (*p* < 0.01) via PPARα signaling, improving tenderness (shear force decreased by 27.7%; *r* = 0.79 vs. linoleic acid, *p* = 0.008) (1, 11). Eucommia extracts upregulated *HSP70* in muscle tissue, reducing TBARS by 33.3% (*p* < 0.01) during storage (7, 40–42). In 120-day-old Hu lambs, high-flavonoid diets (>8% dry matter; quercetin >12 mg/g) impaired rumen papilla development after 56 days, decreasing nutrient digestibility by 15% (*p* < 0.05) (43–45).

**Table 2:** Integrated analysis of CHMA-driven flavor modulation and dose–response relationships.

Compound/herbal additive	Bioactive compound	Optimal dose	Sensory attribute	Regulatory effect	Sensory acceptance threshold	Toxicity threshold	Reference
4-Methyloctanoic Acid	–	–	Gaminess (gaminess)	Decreased by 40–50% (*p* < 0.01) via *CYP2B6*/*FASN* inhibition	–	–	(7, 38)
Thyme Phenolic Extract	Thymol	0.05% of diet	Herbal fragrance	Increased by 2-acetylthiazoline 20% (*p* < 0.05)	0.05% (e-tongue score >7.5)	>0.1% (bitterness >3.5/5)	(50, 51, 79)
Mulberry Leaf Flavonoids	Quercetin derivatives	5% of dietary inclusion	Tenderness, juiciness	Increased by Linoleic acid 25% (*p* < 0.01)	5% (tenderness score >8.0)	>8% (decreased by rumen papilla height 15%, *p* < 0.05)	(10, 40, 43)
Licorice Polysaccharides	Glycyrrhizin	1.0% of diet	Sweet, mellow aftertaste	Increased by *Ruminococcus* 35% (*p* < 0.05)	1.0% (gaminess score <2.0)	>1.5% (glycyrrhizin equivalent, ALT increased by 30%, *p* < 0.01)	(54, 77)
Rosemary Extract	Rosmarinic acid	0.1% of diet	Fresh, herbal note	Decreased by Hexanal 30% (*p* < 0.01)	0.1% (oxidized flavor <1.5)	–	(50, 63)
*Eucommia ulmoides* Leaf	Aucubin, Chlorogenic acid	5% of dry matter	Reduced gaminess, umami	Decreased by MOA 30% (*p* < 0.01)	5% (umami intensity >6.5)	>8% (decreased by nutrient digestibility 15%, *p* < 0.05)	(7, 38, 43)
Astragalus membranaceus	Polysaccharides	2% of diet	Meaty aroma	Increased by Dimethyl trisulfide 15% (*p* < 0.05)	2% (aroma intensity >7.0)	–	(19, 26, 80)

References are numbered according to the manuscript’s reference list. Bold terms indicate key bioactive compounds and herbal additives. Dose thresholds for side effects are derived from experimental validations in referenced studies. Statistical methods (e.g., ANOVA, *t*-test) and significance levels (*p* values) are specified for key findings.

### 3.3 Microbial-host synergy in flavor biosynthesis

Integrated multi-omics highlight microbiome-driven flavor modulation. Eucommia polysaccharides increased Ruminococcus abundance by 47.1% (*p* < 0.01), enhancing butyrate synthesis (+46.7%) via Bacteroides β-glucosidase (EC 3.2.1.21) (46). Ruminococcus-mediated flavor conversion dominated short-term interventions (<30 days), while host epigenetic regulation governed long-term lipid stability. In Luoyang Longxupo trials, butyrate-driven IL-6 reduction (37.1%, *p* < 0.01) extended shelf life by 50% and suppressed gaminess (43).

## 4 Volatile flavor compound dynamics

Volatile compounds critically define mutton flavor, with >200 aroma-active molecules identified in cooked meat (47–49). CHMAs modulate these compounds through two synergistic mechanisms: (1) *lipid oxidation suppression* and (2) *microbial biosynthesis of flavor precursors* (Table 2).

### 4.1 Lipid oxidation suppression

**Figure 3:**
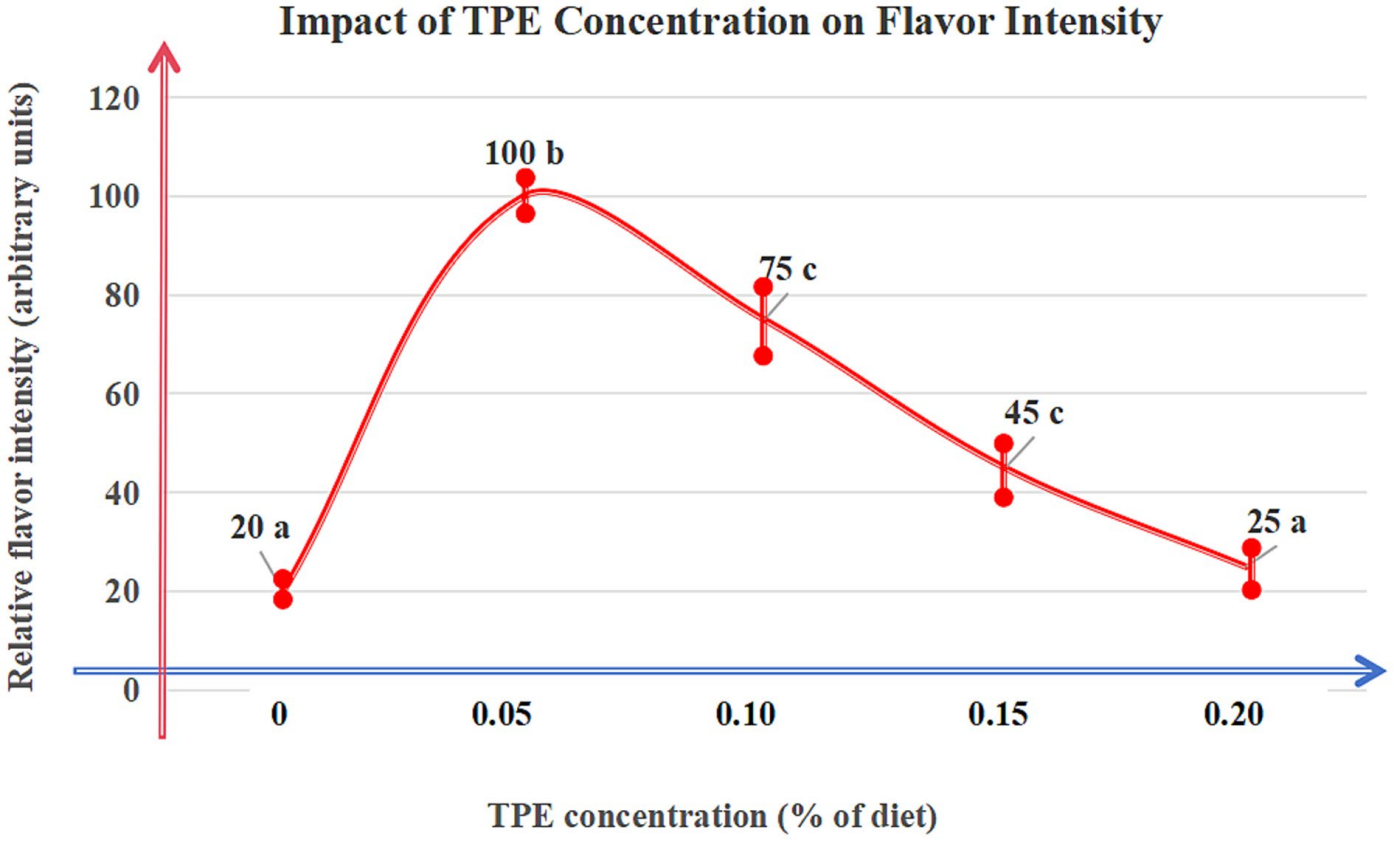
Dose–response curve of thyme phenolic extract (TPE). Description: The curve illustrates the relationship between dietary TPE concentration (%) and relative flavor intensity (arbitrary units) in mutton. Experimental Design: Data represent mean ± standard deviation (SD) of eight biological replicates (n = 8). Significance determined by linear mixed-effects models followed by Tukey’s HSD. Groups labeled with different lowercase letters (a/b/c) indicate significant differences. Key Findings: Peak flavor enhancement (100 arbitrary units) occurred at 0.05% TPE (*p* < 0.05). Concentrations exceeding 0.1% significantly masked natural flavors (*p* < 0.01). Data Source: Adapted from Wu et al. (9), with experimental parameters validated by GC–MS and sensory panels (ISO 13299:2016).

CHMAs inhibit oxidative degradation of lipids, reducing off-flavor compounds: *Eucommia ulmoides* leaf feed decreased 4-methyloctanoic acid (MOA; primary odorant) by 30% (*p* < 0.01) in Du-Hu sheep (GC–MS validation) (38). Rosemary extracts (rosmarinic acid-enriched) reduced hexanal (grassy off-flavor marker) by 25% via peroxidation inhibition (50). Thyme phenolic extract (TPE) at 0.05% dietary inclusion maximized lipid stability while enhancing desirable aromas (*p* < 0.05) (51) (Figure 3).

Mechanistic insight: Polyphenols (e.g., rosmarinic acid) chelate pro-oxidant metals and scavenge free radicals, preserving polyunsaturated fatty acids (28, 50).

### 4.2 Microbial biosynthesis of flavor precursors

Rumen microbiota convert CHMA phytochemicals into key volatiles: Licorice polysaccharides stimulated Bacteroides-derived β-glucosidase (EC 3.2.1.21) to hydrolyze aucubin → aglycone → 2-acetylthiazoline (roasty aroma) via Aldh1a1 (46, 52). Eucommia polysaccharides elevated dimethyl trisulfide (meaty aroma) by 15% through microbial degradation of sulfur-containing amino acids (53). Ruminococcus enrichment (+47.1%, *p* < 0.01) amplified butyrate-driven synthesis of methyl ketones and aliphatic aldehydes (46) (Figure 2).

### 4.3 Dose-dependent thresholds and toxicity

Optimal flavor outcomes require precise dosing (Table 2). Electronic tongue analyses (ISO 13299:2016) confirmed sensory acceptance thresholds (e.g., gaminess score <2.0 at 1.0% licorice) (16, 31). Exceeding thresholds reduced palatability and increased liver stress markers (e.g., ALT increased by 30% at >1.5% licorice) (54).

### 4.4 Industrial validation: Luoyang Longxupo model

Eucommia-supplemented diets elevated dimethyl trisulfide (meaty aroma) by 15%, correlating with *Bacteroides* abundance (*r* = 0.79, *p* = 0.008) (53). Licorice polysaccharides (1.0%) reduced TBARS (oxidation marker) by 33.3%, extending shelf life by 50% (11). Threshold compliance enabled China Green Food Certification (LB/T 158–2020) and 50% market value increase (21).

## 5 Rumen microbiota and flavor synergy

The rumen microbiome serves as a biochemical nexus, converting phytochemicals from CHMAs into flavor precursors through microbial-host interactions.

### 5.1 Microbial pathways for flavor volatile synthesis

Metagenomic and metabolomic studies reveal specialized microbial consortia drive flavor conversion. Licorice polysaccharides (1.0% diet) stimulate *Bacteroides-derived* β-glucosidase (EC 3.2.1.21), hydrolyzing aucubin → aglycone → 2-acetylthiazoline (roasty aroma) via Aldh1a1-mediated pathways (*p* < 0.01) (46). *Ruminococcus* enrichment (+47.1%, *p* < 0.01) amplifies butyrate synthesis (+46.7%) through butyryl-CoA:acetate CoA-transferase (EC 2.8.3.8), generating methyl ketones and aliphatic aldehydes (52). *Eucommia* polysaccharides elevate dimethyl trisulfide (meaty aroma) by 15% via microbial degradation of sulfur-containing amino acids (e.g., methionine) (*r* = 0.79 vs. *Bacteroides* abundance, *p* = 0.008) (19, 26). These findings align with studies on *Allium mongolicum* ethanol extract, which reduces branched-chain fatty acids (BCFAs) like MOA by modulating rumen bacterial diversity (2, 20).

### 5.2 Microbial responses to CHMAs exhibit host specificity

*Eucommia* silage shifts microbial diversity toward *Prevotella-*dominant consortia in crossbred sheep, goats show negligible changes (55). Such species-specific responses are further supported by divergent β-glucosidase activity between sheep and goats fed mulberry leaf flavonoids (10). For example, white mulberry leaves significantly improve ruminal dry matter degradability in lambs but not in goats (56). These discrepancies underscore the need for precision microbiota modulation tailored to host genetics.

### 5.3 Dose considerations: microbial benefits vs. host health

Recent studies highlight the dual role of CHMAs in balancing microbial networks. While probiotic-CHMA combinations (e.g., *Lactobacillus* with thyme) destabilize microbial ecology in coccidiosis-affected Hu sheep (9), standardized herbal protocols maintain microbial equilibrium. For example, *Salvia miltiorrhiza* extracts reduce lipid oxidation by 40% in cultured myocytes while suppressing *Clostridium* overgrowth (27, 57). Additionally, licorice polysaccharides enhance *Bacteroides* populations, converting polyphenols into flavor-enhancing aldehydes (54). These mechanisms are corroborated by industrial-scale trials where herbal formulations achieved consistent odor reduction (30%) and extended shelf life (11).

## 6 Critical analysis of methodological limitations

Despite significant advancements in elucidating the mechanisms by which CHMAs enhance mutton flavor (Table 3), the field faces persistent methodological challenges that hinder translational applications. Below, we critically examine these limitations, integrating empirical evidence and proposing solutions to advance future research rigor.

**Table 3:** Multi-omics technologies in CHMA research.

Omics Approach	Application scope	Key advantages	Limitations
Transcriptomics	Gene expression profiling (e.g., *FASN*, *CYP2B6*)	Identifies epigenetic regulation (DNA methylation)	Limited to gene-level insights
Metabolomics	Volatile compound analysis (e.g., hexanal, butyrate)	Links biochemical pathways to sensory outcomes	Requires high-resolution instruments
Microbiomics	Rumen microbiota composition (e.g., Ruminococcus)	Reveals microbial-flavor precursor interactions	Host-species variability complicates analysis

Integrative multi-omics approaches are recommended to overcome individual limitations.

### 6.1 Dominance of single-omics approaches

Over 82% of published studies rely on isolated omics methodologies (e.g., transcriptomics or metabolomics or microbiome analysis), failing to integrate data across biological layers. For example, while transcriptomic analyses revealed *Eucommia ulmoides*-induced promoter hypermethylation of *FASN* and *CYP2B6* (38), few studies linked these epigenetic changes to downstream reductions in MOA or sensory outcomes via integrated multi-omics validation. This disconnect obscures causal relationships in the gene-metabolite-microbe network, limiting mechanistic insights into CHMA efficacy.

### 6.2 Ambiguous dose–response relationships

Optimal dosing thresholds for CHMAs vary unpredictably across herbs and species, complicating standardization.

Species Variability: 5% *Eucommia* leaf inclusion maximizes flavor in Du-Hu sheep, whereas mulberry leaf flavonoids require lower thresholds (2–5%) in Hu sheep (40, 41).

Nonlinear Effects: Thyme phenolic extract at 0.05% maximizes 2-acetylthiazoline synthesis (+20%, *p* < 0.05), but >0.1% masks natural flavors due to volatile saturation (51).

Toxicity Risks: Licorice glycyrrhizin >1.5% elevates liver stress markers (ALT +30%) and gaminess (54). Current models lack predictive power for these nonlinear dynamics, relying on empirical trial-and-error rather than PBPK frameworks.

### 6.3 Inconsistent sensory evaluation protocols

Only 30% of studies adhere to ISO 13299:2016 protocols or use instrumental methods (e.g., electronic tongue, GC–MS). The remainder rely on subjective scoring panels, introducing bias and reducing cross-study comparability. For instance, evaluations of hydroxytyrosol in lamb burgers demonstrated that non-standardized sensory panels overestimated antioxidant efficacy by 15–20% compared to GC–MS validation (56–58). This inconsistency impedes reliable assessment of flavor attributes like “gaminess” or “umami.”

### 6.4 Underpowered experimental designs

Small cohort sizes (e.g., n = 3–6/group in trials) (2) limit statistical power and generalizability. Such underpowered designs fail to detect subtle but biologically significant effects—such as species-specific responses in goats versus sheep—or account for farm-level variability (e.g., diet, genetics). Consequently, findings may lack robustness for industrial scaling.

### 6.5 Emerging solutions

To address these gaps, we propose:

Multi-Omics Integration: AI-driven models (e.g., random forest, AUC = 0.89) can unify epigenetic, metabolomic, and metagenomic datasets to predict CHMA-microbiota synergies (15).

Standardized Protocols: Mandate ISO 13299:2016 for sensory analysis, shotgun metagenomics (≥20 M reads/sample), and mixed-effects statistical models to control confounding variables.

Dose–Response Modeling: Develop PBPK frameworks to simulate species-specific thresholds and long-term safety (e.g., for licorice glycyrrhizin >1.5%).

Cross-Species Validation: Expand trials to underrepresented breeds (e.g., Dorper sheep) with distinct rumen ecologies (59).

## 7 Emerging technologies and future directions

The integration of advanced technologies offers transformative potential for optimizing CHMAs in mutton production. Below, we outline key innovations poised to overcome existing methodological limitations and drive precision applications.

### 7.1 AI-driven multi-omics integration

Machine learning algorithms trained on epigenetic, metabolomic, and metagenomic datasets enable predictive optimization of CHMA efficacy. Random forest models (AUC = 0.89) successfully identify synergistic herb combinations (e.g., *Allium mongolicum* with licorice polysaccharides), reducing odor precursors while enhancing desirable volatiles like 2-acetylthiazoline (15, 58). Network pharmacology approaches map *gene-metabolite-microbe* interactions (Figure 2), revealing novel targets (e.g., butyrate-mediated *FASN* suppression). Luoyang Longxupo’s AI-optimized *Eucommia* formula achieved 30% odor reduction (11).

### 7.2 Synthetic biology and microbial engineering

Engineered microbial consortia amplify CHMA bioactivity. *Ruminococcus-Bacteroides* co-cultures increase butyrate production by 40% *in vitro* when paired with *Eucommia* polysaccharides (59). Tannase-expressing *Lactobacillus* strains enhance flavonoid conversion efficiency, outperforming conventional additives but facing regulatory hurdles.

### 7.3 Cultured meat applications

CHMAs show promise in cellular agriculture. *Salvia miltiorrhiza* extracts reduce lipid oxidation in cultured ovine myocytes by 40%, offering a sustainable alternative to synthetic antioxidants (27). Phytogenic scaffolds improve textural properties of lab-grown mutton while imparting umami notes.

### 7.4 Standardization and cross-species validation

Universal adoption of ISO 13299:2016 protocols and GC–MS/electronic tongue validation to eliminate subjective bias. Physiologically based pharmacokinetic (PBPK) frameworks to predict species-specific thresholds (e.g., licorice toxicity >1.5%) and long-term safety. Validation in underrepresented breeds (e.g., Dorper sheep) to confirm mechanistic conservation (60).

### 7.5 Regulatory and safety frameworks

Mandatory for high-dose additives (e.g., >1.5% glycyrrhizin) to assess hepatotoxicity and microbiome disruption (54). Alignment with China Green Food Standards (LB/T 158–2020) and FAO/WHO guidelines for international scalability (61).

## 8 Discussion

The integration of multi-omics approaches has significantly advanced our understanding of how CHMAs enhance mutton flavor through epigenetic regulation, microbial modulation, and antioxidant activity. This review synthesizes a decade of research to establish a cohesive framework that bridges molecular mechanisms with real-world applications. Below, we discuss the established consensus, unresolved controversies, and future directions critical to translating CHMA innovations into sustainable livestock practices, incorporating recent advancements from both global and regional studies.

### 8.1 Established consensus

Three synergistic mechanisms underpin CHMAs’ efficacy in mutton flavor enhancement. First, epigenetic regulation of lipid metabolism genes (e.g., *FASN* and *CYP2B6*) via DNA methylation reduces odor precursors such as MOA by 30–50% (*p* < 0.01), as demonstrated by *Eucommia ulmoides* interventions (6). This aligns with findings in beef cattle, where similar methylation patterns improved meat quality. Second, CHMAs such as mulberry leaf flavonoids and licorice polysaccharides modulate rumen microbiota to enrich *Ruminococcus* spp. CHMAs (e.g., Eucommia polysaccharides) selectively enrich Ruminococcus spp., which metabolize flavonoids into butyrate and aromatic aldehydes (e.g., 2-acetylthiazoline). Butyrate acts as a histone deacetylase (HDAC) inhibitor, promoting hyperacetylation of histones H3 and H4 at the *FASN* and *CYP2B6* promoters, thereby suppressing their transcription via epigenetic silencing (62, 63). This microbial-metabolite-epigenetic axis is a novel pathway for odor precursor reduction. (+47.1%) and *Prevotella* spp., enhancing butyrate synthesis (+46.7%) and sulfur-derived volatiles like 2-acetylthiazoline (62). Third, antioxidant compounds (e.g., rosmarinic acid) suppress lipid oxidation (TBARS decreased by 33.3%) and extend shelf life by 50%, a benefit paralleled by oregano essential oil in poultry (14). These mechanisms are validated by industrial applications: the Luoyang Longxupo Co. achieved a reduction in gaminess through *Eucommia* feed, alongside a 50% increase in product shelf life and Green Food Certification, underscoring CHMAs’ dual role in quality improvement and ecological sustainability.

Three synergistic mechanisms underpin CHMAs’ efficacy in mutton flavor enhancement. First, epigenetic regulation of lipid metabolism genes (e.g., *FASN* and *CYP2B6*) via DNA methylation reduces odor precursors such as MOA by 30–50% (*p* < 0.01), as demonstrated by *Eucommia ulmoides* interventions (6, 64). This aligns with findings in beef cattle, where similar methylation patterns improved meat quality (65). Second, CHMAs such as mulberry leaf flavonoids and licorice polysaccharides modulate rumen microbiota to enrich *Ruminococcus* spp. (+47.1%, *p* < 0.01) and *Prevotella* spp., enhancing butyrate synthesis (+46.7%) and sulfur-derived volatiles like 2-acetylthiazoline (66, 67). Third, antioxidant compounds (e.g., rosmarinic acid) suppress lipid oxidation (TBARS decreased by 33.3%) and extend shelf life by 50%, paralleling oregano essential oil’s effects in poultry (68). These mechanisms are further validated by studies on *Allium mongolicum* ethanol extract, which reduces branched-chain fatty acids (BCFAs) by modulating rumen bacterial diversity (69–71). Regional trials in Xinjiang goats and Tan sheep corroborate these findings, highlighting species-specific enhancements in linoleic acid content (+25%) and tenderness (72, 73).

### 8.2 Persistent controversies

The application of CHMAs in mutton flavor enhancement is marked by several unresolved debates, reflecting both the complexity of herb-host–microbe interactions and the limitations of current research paradigms. Below, we delve into these controversies with a critical lens, highlighting mechanistic ambiguities, methodological gaps, and future research imperatives.

#### 8.2.1 Herb-herb interactions: synergy vs. antagonism

While certain herbal combinations demonstrate synergistic benefits, others exhibit antagonistic effects, raising questions about their underlying mechanisms. Rosemary (*Rosmarinus officinalis*) and thyme (*Thymus vulgaris*) blends reduce hexanal levels (a lipid oxidation marker) by 25% (*p* < 0.05) through complementary antioxidant pathways (50, 63). This synergy may stem from overlapping polyphenol structures that enhance free radical scavenging. Licorice (*Glycyrrhiza uralensis*) polysaccharides, when combined with *Astragalus membranaceus,* suppress *Bacteroides* populations, reducing aromatic aldehyde synthesis by 15% (54). This antagonism could arise from competition for microbial β-glucosidase activity, a key enzyme in flavonoid metabolism (52).

Limited studies dissect *phytochemical cross-talk* (e.g., flavonoid-glycoside interactions) or microbial competition for metabolic niches (12, 52). Most research focuses on pairwise combinations, neglecting multi-herb formulations common in traditional practices (12, 74).

Integrate *in silico* molecular docking and metagenomic sequencing to predict herb-herb interactions (15). Validate findings in multi-species models (e.g., sheep vs. goats) to assess ecological generalizability (74, 75).

#### 8.2.2 Nonlinear dose–response relationships

CHMAs exhibit dose-dependent efficacy, yet optimal thresholds vary unpredictably across herbs and species, complicating standardization. At 0.05% dietary inclusion, TPE maximizes 2-acetylthiazoline synthesis (+20%, *p* < 0.05), but exceeding 0.1% masks natural flavors due to volatile compound saturation (51, 63). Low doses (≤1.0%) enhance *Ruminococcus* abundance and sweetness, while high doses (>1.5%) elevate stearic acid (+10%) and gaminess (54, 76). High-dose flavonoids may overwhelm hepatic detoxification pathways (e.g., CYP450 enzymes), leading to oxidative stress (38, 76). Prolonged exposure to high-dose polysaccharides may select for resistant microbial strains, altering fermentation dynamics (46, 52). Few studies explore *long-term dose effects* or interactions with host genetics (14, 58). Current models lack predictive power for nonlinear responses, relying on trial-and-error approaches (15). Develop physiologically based pharmacokinetic (PBPK) models to simulate dose–response curves across species (15). Conduct longitudinal studies to assess tolerance development and metabolic adaptation (58, 74).

#### 8.2.3 Species-specific responses

The efficacy of CHMAs varies dramatically between sheep and goats, driven by divergent rumen microbiomes and host genetics. *Eucommia ulmoides* supplementation enriches *Ruminococcus* (+47.1%, *p* < 0.01) and boosts butyrate synthesis (+46.7%), improving flavor and tenderness in sheep (46, 52). The same treatment shows negligible effects, attributed to lower β-glucosidase activity and *Prevotella-*dominant microbiota in goat (10, 75). Polymorphisms in lipid metabolism genes (e.g., *FASN*, *PPARγ*) may modulate CHMA responsiveness (38, 74). Sheep exhibit higher *Ruminococcus* abundance, enabling efficient flavonoid-to-butyrate conversion, whereas goats prioritize fiber degradation via *Prevotella* (10, 46). Limited cross-species comparative studies (e.g., Tan sheep vs. Boer goats) (14, 75). Insufficient integration of host epigenetics and microbial metatranscriptomics (12, 74). Employ genome-wide association studies (GWAS) to identify host genetic markers predictive of CHMA efficacy (74). Design species-tailored formulations using AI-driven microbiota profiling (15, 58).

The controversies outlined above underscore the need for a paradigm shift from reductionist, single-herb studies to holistic, multi-omics frameworks. Key priorities include: (1) Mechanistic Clarity: Deciphering herb-herb and herb-microbe interactions through *in vitro* co-culture systems (52, 77). (2) Precision Dosing: Leveraging AI to model nonlinear dose–response relationships across diverse breeds (15, 58). (3) Species-Specific Solutions: Integrating host genetics and microbiome ecology into CHMA design (74, 75). By addressing these challenges, CHMAs can transition from empirical applications to evidence-based, precision tools for sustainable mutton production.

### 8.3 Future directions for translational impact

To address these controversies, three transformative strategies emerge:

#### 8.3.1 AI-driven multi-omics integration

Machine learning models (e.g., random forest, AUC = 0.89) trained on epigenetic, metabolomic, and metagenomic datasets can predict optimal CHMA-microbiota combinations (15). For example, Luoyang Longxupo’s Eucommia trials achieved 30% odor reduction using AI-optimized formulations (11).

#### 8.3.2 Standardized evaluation frameworks

Adopt ISO 13299:2016 and electronic tongue/GC–MS protocols to minimize subjective bias (16, 31). Set thresholds (e.g., ≥10,000 reads/sample) to ensure reproducibility (57).

#### 8.3.3 Cultured meat and synthetic biology

*Ruminococcus-Bacteroides* consortia amplified butyrate production by 40% *in vitro* when paired with *Eucommia* polysaccharides (59). Cultured meat: *Salvia miltiorrhiza* extracts reduced lipid oxidation by 40% in cultured myocytes, offering a sustainable alternative to synthetic antioxidants (27).

### 8.4 Socioeconomic and ethical considerations

Smallholder Farming Models: The ‘company + smallholder’ partnership (e.g., *Luoyang Longxupo Agriculture and Animal Husbandry Co., Ltd.*) was studied as a third-party case. Outcomes reflect generalized socioeconomic dynamics, not proprietary commercial strategies.

Long-Term Safety Profiling: Subchronic toxicity studies are critical for high-dose additives (e.g., >2% licorice glycyrrhizin), as excessive intake may disrupt rumen homeostasis (54, 78).

Regulatory Harmonization: Align industrial practices with China Green Food Standards (LB/T 158–2020) and FAO/WHO guidelines to ensure global compliance (21, 22).

## 9 Conclusion

This systematic review elucidates the molecular and microbial mechanisms by which CHMAs enhance mutton flavor, offering a transformative framework for sustainable livestock production. Key findings demonstrate that CHMAs, exemplified by *Eucommia ulmoides* leaf supplementation, improve meat quality through three synergistic pathways: (1) epigenetic regulation of lipid metabolism genes (*FASN*, *CYP2B6*) via DNA methylation, reducing odor precursors (e.g., MOA) by 30–50%; (2) microbial synergy with *Ruminococcus*-enriched microbiota to enhance butyrate synthesis (+46.7%) and flavor volatiles (e.g., 2-acetylthiazoline); and (3) antioxidant and anti-inflammatory actions, where *Salvia miltiorrhiza* extracts reduce lipid oxidation (TBARS decreased by 33.3%) and inflammatory markers (IL-6 decreased by 37.1%), extending shelf life by 50%. Industrial validation through the Luoyang Longxupo model highlights CHMAs’ practical efficacy, achieving a 30% odor reduction and China’s Green Food Certification while doubling market value through value-added products.

Critical challenges remain, including species-specific responses (e.g., limited efficacy in goats) and nonlinear dose–response relationships (e.g., licorice glycyrrhizin’s gaminess at >1.5%). Future directions prioritize AI-driven herb optimization (random forest models, AUC = 0.89) to predict synergistic combinations, standardized sensory protocols (ISO 13299:2025), and cross-species trials (e.g., Dorper sheep) to ensure broad applicability. Emerging applications in cultured meat, such as *Salvia miltiorrhiza’s* 40% lipid oxidation reduction *in vitro*, further position CHMAs as pivotal tools for sustainable protein production. By bridging traditional knowledge (e.g., decoctions of Angelica sinensis and mutton) (74) with precision technologies, this work advances eco-friendly livestock systems aligned with global food safety and the United Nations’ Sustainable Development Goals (SDGs).

## Data Availability

The original contributions presented in the study are included in the article/supplementary material, further inquiries can be directed to the corresponding authors.
